# Long-term cover cropping seasonally affects soil microbial carbon metabolism in an apple orchard

**DOI:** 10.1080/21655979.2019.1622991

**Published:** 2019-06-06

**Authors:** Jianfeng Yang, Tairan Zhang, Rongqin Zhang, Qianqian Huang, Huike Li

**Affiliations:** a College of Natural Resources and Environment, Northwest A&F University, Yangling, Shaanxi, China; b Key Laboratory of Plant Nutrition and the Agro-Environment in Northwest China, Ministry of Agriculture, Yangling, China

**Keywords:** Biolog, living mulch, microbial metabolism, apple orchard, season

## Abstract

Groundcover management can significantly affect soil microbial metabolic activities, especially carbon metabolism, in apple orchards. However, there have been few studies on the effects of groundcover on the seasonality of soil microbial carbon metabolism. We, therefore, studied soil microbial carbon metabolism in an apple orchard on China’s Loess Plateau under four single species cover crops (the grass *Dactylis glomerata* L., and the legumes *Trifolium repens, Coronilla varia* L., *Lotus corniculatus* L.) during spring, summer and fall. Cover cropping significantly, but differentially, promoted soil microbial carbon metabolism in spring and fall. However, cover cropping leads to a significant reduction of soil moisture in spring and summer due to the competition of soil moisture between the cover crops and apple trees, which probably lead to the changes in types of carbon substances metabolizing by soil microbes in summer. Besides, cover crop significantly enhanced soil organic carbon contents between three seasons while clean cultivation had slight, non-significant effects. The promotion of soil microbial metabolic activities was probably an important mechanism for the carbon accumulation, and we postulate that leguminous cover plants may have significantly different effects, mediated through their root exudates, from grasses on soil carbon contents.

## Graphical Abstract

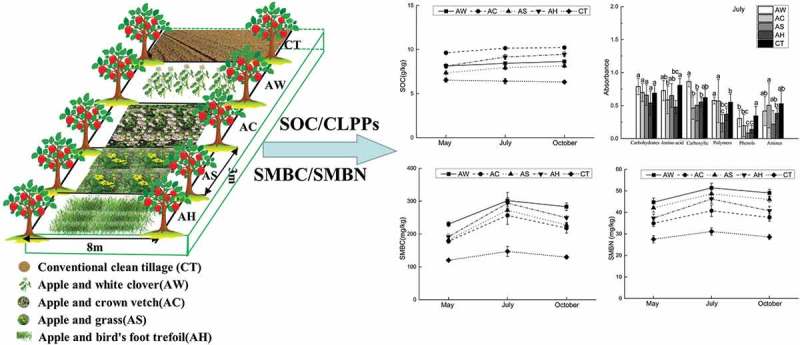

## Introduction

Cover crops have proven utility in conferring benefits to soil with respect to organic matter and allied benefits [1]. In recent years, they have gained considerable popularity within annual cropping, but are underused within perennial systems. Strategies of this kind, also known as living mulch or groundcover management systems, can have strong, sustained beneficial effects on the chemical properties of soil and microbial communities [2]. On the Loess Plateau of China, where there have been substantial soil organic matter losses, nutrient imbalances and moisture deficits, mulching using cover crops is important for local economies and the environment [3]. This region includes one of the largest apple production areas in China, which produces very high-quality fruits. Mulching with cover crops such as white clover (*Trifolium repens* L.) and crown vetch (*Coronilla varia* L.) that are adapted to the local climate and soil conditions has been used for decades in this region’s orchards [4].

Many studies have explored the effects of various soil management regimes on soil microbial communities, because microbes are among the most active and important components of any soil ecosystem [5]. These studies have shown that soil management strategies based on mulching or cover crops can induce highly beneficial changes in the composition and behavior of the soil microbial community [6,7]. The effects of cover crops on soil microorganisms can be measured by considering parameters such as the diversity of the microbial community and microbial metabolic activities. Many studies conducted in China have demonstrated increases in soil microbial diversity following the introduction of cover crops [8]. However, most of these studies did not extend over multiple seasons; generally, they examined the composition of the microbial community in specific months relevant to certain problems. For example, Qian et al. studied phenomena in June while other groups focused on communities in September. Few authors have investigated relevant seasonal changes. A notable exception is that measured microbial metabolic activities and characterized a functional microbial community under two land-use (apple orchard and boundary bush) over 2 years in spring, summer and fall [9]. Their results revealed significant differences in results of the studied land-use type in the first, but not the second year.

Soil microbial carbon metabolism is an important indicator of soil quality, and the Biolog microplate technique has been widely used to measure capacities of microorganisms to utilize different carbon substrates in analyses of functional diversity in soil microbial communities [10]. Although assessments of functional diversity using this technique are one-sided [11], it is useful for comparing the functional ability of entire soil microbial communities in contrasting environmental samples [12]. This method was recently used to analyze microbial carbon metabolism [13], but few researchers have used it to study microbial carbon metabolism under living mulches.

To address the knowledge gaps discussed above, we evaluated soil microbial carbon metabolic capacity in an apple orchard three times per year (May, July and October, which reflected three local seasons spring, summer and fall, respectively) after 10 years of interplanting with cover plants to determine how seasonal changes affect soil microbial metabolism under groundcover management in the long term. Plots planted with four single cover plants (three legumes and one grass) were monitored to determine how different cover crops affect the seasonality of microbial carbon metabolic capacity. In addition, the soil organic carbon (SOC), soil microbial biomass carbon, nitrogen (SMBC/SMBN) and moisture contents were measured to explore possible reasons for differences in soil microbial metabolic activities under different groundcover regimes. We hypothesized that: (1) soil microbes under different cover crops would exhibit different trends in carbon metabolic activity; (2) these trends would exhibit seasonal variation reflecting changes in the cover crops’ physiological activities.

## Materials and methods

### The study site and treatments

The experiment was conducted at the Northwest A&F University Apple Experimental Station, Baishui county (35°21′N, 109°30′W; Elevation 850 m), Weibei Loess Plateau, Shaanxi Province, China. The orchard’s soil is a very homogeneous silty clay loam, and the site has been intensively cultivated over several centuries. The soil is a loessial soil and was classified as a Calcic Cambosol according to the Chinese Soil Taxonomy [14]. Before sampling, the orchard’s soil had a pH of 8.46, and total organic carbon, nitrogen, phosphorus and potassium contents of 7.82, 0.46, 0.50 and 16.28 g kg^−1^ (under clean cultivation (CT)), respectively. The local average annual rainfall amounts to 577.8 mm, with over 80% falling between June and September. The site’s average annual temperature is 11.4°C, with large yearly fluctuation. The climate was conducive to producing high-quality apples. The apple trees used in this study were of ‘Fuji’ cultivar, grafted on M26 rootstock (*Malus ×domestica* cv.). The trees were 15 years old at the start of the sampling, and were planted at intervals of 3 m between rows and 8 m between lines.

Four treatments were initiated following clean CT and have been sustained (together with the continuation of clean cultivation, CT, as a control treatment) since March 2005 using a randomized complete block design with three replicates per treatment. The treatments are: (1) AW – intercropping of apple and white clover (*Trifolium repens*); (2) AC – intercropping of apple and crown vetch (*Coronilla varia* L.); (3) AS – intercropping of apple and the grass (*Dactylis glomerata* L.); and (4) AH – intercropping of apple and bird’s foot trefoil (*Lotus corniculatus* L.). Seeds of the cover plants were sown in 4 m strips between adjacent tree rows at 0.75 g/m^2^ after deep tillage. Each experimental plot covers an area of 30 m^2^ and contains six apple trees. The grasses were mowed 2–3 times per year, and the clean CT plots were weeded four times a year. Fertilization and pest control were performed as required to maintain the health and growth of the apple trees.

### Sampling

Nine apple trees with homogeneous growth vigor were selected in each plot and marked at the beginning of the experiment in March 2005. In each season (May, July and October of 2015), three different trees were chosen in each plot to get soil samples. Ten soil samples (five of the 0–20 cm layer and five of the 20–40 cm layer) were collected from randomly distributed spots within a 1 m radius beneath cover crops around the marked trees in each treatment plot after clearing surface litter. After removing stones, visible root pieces and un-decomposed organic matter by passage through a 2 mm mesh sieve all soil samples from the same plot (*n* = 30) were homogenized and subdivided. The first subsamples were placed in a sterile plastic bag, labeled, sealed and transported to the laboratory on ice and finally stored in plastic bags at 4°C until needed for Biolog analysis, which was performed within 24 h, and for the SMBC/SMBN determination, which was performed within 2 days. The second subsamples were air-dried first before SOC determination.

### Community-level physiological profiles (CLPPs)

In this work, 5 g (dry weight) subsamples of the pooled orchard soil samples were suspended in 45 mL of sterile saline (0.85% NaCl) and shaken at 120 rpm for 30 min to obtain CLPPs using a Biolog Microstation™ (BIO-TEK Instruments Inc., Winooski, VT, USA). Each supernatant was diluted to 10^−3^ with sterile saline, and 150 mL of the diluted supernatant was placed in a separate well of a Biolog ECO microplate (Biolog Co., Hayward, CA, USA). The microplates were incubated at 25 ± 1°C, and the absorbance of the mixture in each well at 590 nm was recorded every 24 h for 10 days. Because the absorbance values increased rapidly during the first 120 h of the experiments and then rose slowly between 120 and 240 h, the absorbances recorded after 120 h were used to compute average well color development (AWCD) values and other indices.

SOC levels were determined by the oil bath-K_2_Cr_2_O_7_ titration method following the method [15]. Soil moisture contents were determined gravimetrically by weighing 5–10 g samples of undisturbed soil before and after oven-drying 105°C for 48 h. SMBC and SMBN values were estimated by the chloroform fumigation extraction method [16].

### Data analysis

The CLPPs data were analyzed according to Garland and Mills [17]. AWCD values were computed using the following expression:

$$AWCD=\sum_{i=1}^{n}\left(C_{i}-R\right)/n.$$

Here, *C_i_* is the absorbance of the mixture in well *i* (each filled well on a given microplate contains one of 31 carbon substrates). *R* is the absorbance of a control well, and *n* is the number of carbon substrates included in the assay.

The Shannon index was computed using the following expression [18,19]:

$$H=-\sum \;P_{i}\times \ln P.$$

Here, *P_i_* is the absorbance of the *i*th well relative to the average absorbance for all wells on the plate:

$$P_{i}=\;\;(C_{i}-R)/\sum \left(C_{i}-R\right).$$

The Richness (S) was taken to equal the total number of wells in which the carbon source was utilized by the microbial population, which was indicated by an optical absorbance above 0.25.

The Evenness index was computed as *E* = *H*/ln (*S*), where *H* and *S* are the Shannon and Richness indices, respectively.

The Origin 8.0 software package was used to analyze data and draw figures. The data were analyzed by SPSS 20.0 software package including two-way ANOVA analyses, and the least significant difference test was used to identify significant differences. Pearson correlation coefficients and Path coefficients were used to evaluate relationships between different factors based on one-way ANOVA.

## Results

### Soil moisture

Soil moisture in May and July was significantly lower for living mulch treatments compared to clean CT treatment, while there were no significant differences between under cover crops and CT treatment in October, which are shown in Table 1.

**Table 1.** Soil moisture (means and standard deviations) seasonal dynamics under indicated cover crops.

Treatment	Soil moisture %		
	May	July	October
AW	10.37 ± 0.68 Bc	6.67 ± 0.80 Cc	16.28 ± 0.15 Aa
AC	10.63 ± 0.47 Bc	6.14 ± 0.52 Cd	16.49 ± 0.15 Aa
AS	12.01 ± 0.71 Bb	7.62 ± 0.63 Cb	16.47 ± 0.99 Aa
AH	10.90 ± 0.94 Bbc	7.08 ± 0.06 Cbc	16.25 ± 0.12 Aa
CT	15.86 ± 0.11 Aa	13.93 ± 0.09 Ba	15.92 ± 0.42 Aa

**Note**: Means with different lowercase letters are significantly different between the five treatments at *p* ≤ 0.05 and means with uppercase letters are significantly different between the three times at *p* ≤ 0.05.

(1) AW – intercropping of apple and white clover *(Trifolium repens*); (2) AC – intercropping of apple and crown vetch (*Coronilla varia L*.); (3) AS – intercropping of apple and the grass (*Dactylis glomerata L*.); and (4) AH – intercropping of apple and bird’s foot trefoil (*Lotus corniculatus L*.).

### Soil carbon accumulation

**Figure 1.**
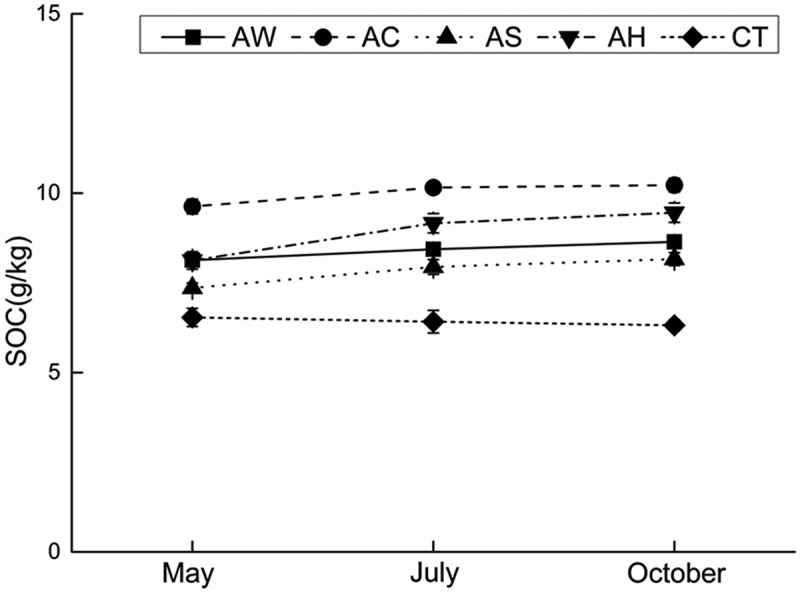
Soil organic carbon contents (mean ± standard deviation) under indicated cover crops. Note: (1) AW – intercropping of apple and white clover (*Trifolium repens*); (2) AC – intercropping of apple and crown vetch (*Coronilla varia L*.); (3) AS – intercropping of apple and the grass (*Dactylis glomerata L*.); and (4) AH – intercropping of apple and bird’s foot trefoil (*Lotus corniculatus L*.).

SOC content significantly increased under the cover crops from May to October (*p*-value: AW 0.001, other <0.001), but did not change under CT treatment during this period (*p*-value: 0.099), showing in Figure 1. However, the increases also varied under different cover crops, especially between AC and the other crops. Increase in SOC was detectable throughout the period not only from May to July but also from July to October under AW (*p*-value: 0.007 and 0.035), AS (*p*-value: <0.001 and 0.018) and AH (*p*-value: <0.001 and 0.020). But the increase under AC was only significant from May to July (*p*-value: 0.001 and 0.445).

### Soil microbial carbon metabolism

The results of Biolog EcoPlates including 31 absorbance change value reflected soil microbial ability to metabolize 31 kinds of measured carbon sources including 10 types of Carbohydrates, 6 types of Amino acids, 7 types of Carboxylic acids, 4 types of Polymers, 2 types of Phenols and 2 types of amines. AWCD is the average of these 31 values and reflects the ability of microbes to metabolize all measured carbon sources, which also could be used to evaluate soil microbial carbon metabolism.

There were significant between-treatment differences in terms of AWCD on the Biolog EcoPlates (Table 2), which represented in significant promotion under AW, AC and AS treatments in May and significant promotion under all four cover crops in October compared to CT treatment. But except for AW treatment, other cover crops decreased AWCD value compared to CT treatment in July, which showed that different cover crops would exert different effects to soil microbial carbon metabolic ability compared to traditional clean CT management and these effects caused by cover crops will change with the seasons.

**Table 2.** Average well color development (means and standard deviations) of samples of soil under indicated cover crops after 120 h incubation.

		AW	AC	AS	AH	CT
Indices	Season	*Trifolium* *repens* L.	*Coronil* *la varia* L.	*Lotuscorn* *iculatus* L.	*Dactylis glomerata* L.	Clean cultivation
AWCD	Spring	0.87 ± 0.06 Aa	0.89 ± 0.06 Aa	0.90 ± 0.04 Aa	0.59 ± 0.01 Bb	0.58 ± 0.04 Ab
	Summer	0.71 ± 0.06 Ba	0.58 ± 0.07 Bbc	0.50 ± 0.04 Ccd	0.47 ± 0.02 Cd	0.65 ± 0.03 Aab
	Fall	0.94 ± 0.05 Aa	0.93 ± 0.05 Aa	0.66 ± 0.02 Bb	0.64 ± 0.02 Ab	0.54 ± 0.08 Ac

**Note**: Means with different lowercase letters are significantly different between the five treatments at *p*≤ 0.05 and means with different capitals are significantly different between the three times at *p* ≤ 0.05.

(1) AW – intercropping of apple and white clover (*Trifolium repens*); (2) AC – intercropping of apple and crown vetch (*Coronilla varia L*.); (3) AS – intercropping of apple and the grass (*Dactylis glomerata L*.); and (4) AH – intercropping of apple and bird’s foot trefoil (*Lotus corniculatus L*.).

There were no significant between-season differences under clean CT treatment, but there were significant between-season differences under cover crops treatments: the AWCD values under cover crops in May and October were significantly higher than in July when there were no significant differences under CT treatment between season, which showed that compared to clean CT, cover crops inhibited soil microbial carbon metabolic ability in July or cover crops did not promote soil microbial carbon metabolic ability as in May and October (Table 2).

**Figure 2.**
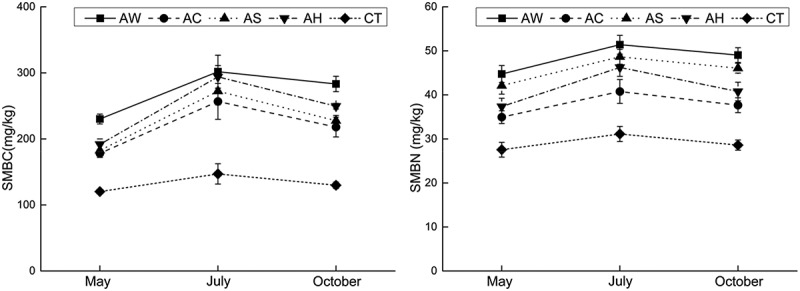
Biolog absorbance readings showing the efficiency of utilization of indicated carbon sources by samples for the soil under indicated covering crops in May, July and October. Note: (1) AW – intercropping of apple and white clover (*Trifolium repens*); (2) AC – intercropping of apple and crown vetch (*Coronilla varia L*.); (3) AS – intercropping of apple and the grass (*Dactylis glomerata L*.); and (4) AH – intercropping of apple and bird’s foot trefoil (*Lotus corniculatus L*.).

Refer to Figure 2: under different cover crops, soil microbes have various utilization ability in using different carbon source types. In May, AW and AS treatments significantly increased soil microbial metabolic capability of Carbohydrates, Amino acids, Polymers, Phenols and Amines when AC treatment only improved soil microbial metabolic capability of Carbohydrates; in July, cover crops significantly decreased soil microbial metabolic capability of Amnio acid, Polymers and Phenols; in October, AC improved soil microbial metabolic capability of Carbohydrates, Amino acids, Carboxylic acids and Polymers and AW improved soil microbial metabolic capability of Carbohydrates, Amino acids and Polymers when AS and AH treatments only improved soil microbial metabolic capability of Amino acids and Polymers, respectively.

Because of the complexity of soil microbial carbon metabolism data, three indices were calculated by the results of Biolog EcoPlates to analyze orchard soil microbial metabolic capacity (Table 3). Shannon diversity showed the diversity of soil microbial carbon metabolism while Richness mainly reflected the number of carbon sources that could be fully used by soil microbes. Evenness can suggest the specialization carbon metabolism of soil microbial. The outcomes of Shannon and Richness indices were similar to the results of AWCD values (Table 3), but there were still some different regulars. In spring, under AC treatment, there were no significant differences compared to CT treatment in Shannon and Richness indices while there were significant differences in Evenness index, which mean that in spring AC treatment promoted the utilization of some specific carbon sources by soil microorganisms, thereby increasing the ability of soil microbial carbon metabolism. And AH did not increase the number of carbon sources that could be fully used by soil microbes in May like AW and AS treatments. But AH treatment did increase Shannon value compared to CT treatment when AWCD in AH and CT treatments did not show any significant differences.

**Table 3.** Diversity indices (means and standard deviations) of soil microbial communities under indicated cover crops.

		AW	AC	AS	AH	CT
Indices	Season	*Trifolium* *repens* L.	*Coronil* *la varia* L.	*Lotuscorn* *iculatus* L.	*Dactylis glomerata* L.	Clean cultivation
Shannon H	Spring	3.04 ± 0.02 Aa	2.63 ± 0.05 Bc	3.10 ± 0.04 Aa	2.75 ± 0.03 Ab	2.57 ± 0.11 Bc
	Summer	2.91 ± 0.05 Ba	2.64 ± 0.12 Bb	2.44 ± 0.06 Cc	2.55 ± 0.15 Bbc	2.94 ± 0.03 Aa
	Fall	3.09 ± 0.02 Aa	3.06 ± 0.03 Aab	2.91 ± 0.04 Bbc	2.93 ± 0.04 Ac	2.77 ± 0.16 ABd
Richness S	Spring	21.33 ± 0.52 Bb	16.50 ± 0.66 Bc	23.33 ± 1.38 Aa	15.83 ± 0.80 Bc	16.00 ± 0.66 Bc
	Summer	19.83 ± 1.28 Ba	13.75 ± 1.75 Cb	13.17 ± 1.01 Cb	13.25 ± 0.75 Cb	19.00 ± 0.50 Aa
	Fall	23.50 ± 0.66 Aa	23.17 ± 0.52 Aa	18.58 ± 0.52 Bb	19.25 ± 0.25 Ab	15.42 ± 2.10 Bc
Evenness E	Spring	1.01 ± 0.02 Aab	0.98 ± 0.00 Bb	0.99 ± 0.01 Bab	1.01 ± 0.01 Aab	1.02 ± 0.04 Ba
	Summer	0.98 ± 0.01 Bb	1.09 ± 0.08 Aa	1.09 ± 0.07 Aa	1.09 ± 0.07 Aa	1.01 ± 0.01 Bab
	Fall	0.98 ± 0.00 Bb	0.98 ± 0.00 Bb	1.02 ± 0.00 Bb	1.06 ± 0.06 Ab	1.19 ± 0.12 Aa

**Note**: Means with different lowercase letters are significantly different between the five treatments at *p* ≤ 0.05 and means with different capitals are significantly different between the three times at *p* ≤ 0.05. Bold type means that there is a significant difference between this treatment and control (CT treatment).

(1) AW – intercropping of apple and white clover (*Trifolium repens*); (2) AC – intercropping of apple and crown vetch (*Coronilla varia L*.); (3) AS – intercropping of apple and the grass (*Dactylis glomerata L*.); and (4) AH – intercropping of apple and bird’s foot trefoil (*Lotus corniculatus L*.).

### Two-way ANOVA and correlation analyses

Because there were two factors that affected soil microbial carbon metabolism, two-way ANOVA analyses were used to distinguish the influences of two factors, season and cover cropping. The outcomes in Table 4 showed that firstly, cover cropping could affect almost all measured microbial carbon metabolism except for Amines and could affect the number of carbon substances that soil microbes metabolized; secondly, time was similar to cover cropping, but it could not affect soil microbes to metabolize Carboxylic acids; finally, there was significant interaction between time and cover cropping on soil microbial carbon metabolism, especially for Carboxylic acids, Phenols and Amines metabolism.

**Table 4.** Summary results of the analysis of variance of effects of practices, time and soil depths on indices of microbial functional diversity and utilization of indicated carbon sources.

Variable	Source	D.f	Sum of squares	Mean square	*F* ratio	Sig.
Treatments	Shannon	0.584	4	0.146	2.331	0.085
	Richness	212.678	4	53.169	7.522	0.000
	Evenness	31.422	4	7.856	9.494	0.000
	AWCD	0.726	4	0.181	10.453	0.000
	Carbohydrates*	1.824	4	0.456	7.468	0.000
	Amino acid*	0.552	4	0.138	3.380	0.025
	Carboxylic acids*	0.493	4	0.123	4.068	0.012
	Polymers*	0.661	4	0.165	3.444	0.023
	Phenols*	0.229	4	0.057	4.124	0.011
	Amines*	0.301	4	0.075	2.391	0.079
Time	Shannon	0.645	2	0.322	5.152	0.014
	Richness	181.693	2	90.846	12.851	0.000
	Evenness	11.640	2	5.820	7.034	0.004
	AWCD	0.393	2	0.197	11.329	0.000
	Carbohydrates*	0.667	2	0.334	5.466	0.011
	Amino acid*	0.959	2	0.479	11.750	0.000
	Carboxylic acids*	0.078	2	0.039	1.281	0.296
	Polymers*	0.424	2	0.212	4.412	0.023
	Phenols*	0.150	2	0.075	5.415	0.011
	Amines*	0.451	2	0.226	7.166	0.004
Treatments × Time	Shannon	1.364	8	0.171	2.724	0.027
	Richness	342.122	8	42.765	6.050	0.000
	Evenness	10.212	8	1.276	1.543	0.195
	AWCD	0.403	8	0.050	2.903	0.021
	Carbohydrates*	0.995	8	0.124	2.037	0.085
	Amino acid*	0.683	8	0.085	2.093	0.077
	Carboxylic acids*	0.634	8	0.079	2.618	0.032
	Polymers*	0.745	8	0.093	1.941	0.100
	Phenols*	0.478	8	0.060	4.304	0.003
	Amines*	0.786	8	0.098	3.121	0.015

* indicates the efficiency of specified carbon sources’ utilization by soil microorganisms.

**Figure 3.**
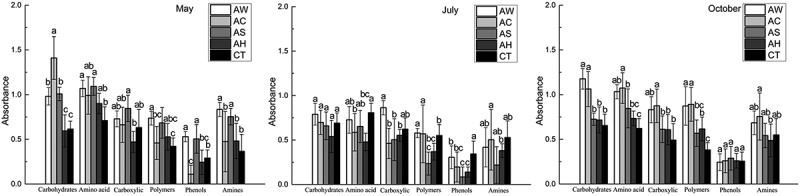
Correlation analysis results in three seasons (solid line means there was a significant correlation between two factors and *p*-value<0.01; dotted line means there was a significant correlation between two factors and *p*-value<0.05).

The correlation analysis reflected the relationship between soil microbial carbon metabolism and other factors showing in Figure 3. There were different correlations in different seasons: in July, local summer, no other measured factor had correlations with AWCD that reflected soil microbial carbon metabolism; in May, almost all measured factors had correlations with AWCD and in October the correlations were similar with in May except for there was no correlation between soil moisture and AWCD.

## Discussion

Many scholars have used Biolog EcoPlates to assess the structure and diversity of soil microbial communities under mulching or cover crops [6–9,20]. However, in recent years, scholars have also begun using them to evaluate the microbial carbon metabolic activity of soil microorganisms. Such investigations can illuminate factors affecting the microbes’ carbon metabolic mechanisms, e.g. salinity [21] and red mud [22]. So, we measured the microbial carbon metabolic capacity under different cover crops. The results in Table 2 and Figure 2 show that the crops can significantly change soil microbial carbon metabolic capacity toward various organic substances, which could reflect carbon metabolic activities of microbes in the orchard soil to some extent. The results also showed that different cover crops would exert different effects to orchard soil microbial metabolic activities, which has been also found in other researches when most of these studies focus on using Biolog Ecoplates data to reflect microbial diversity in topsoil rather than soil microbial carbon metabolism [6].

Although the research focus were different, they also calculated AWCD value like we did like Qian et al. measured AWCD in apple orchard soil under white clover (AW), crown vetch (AC), perennial ryegrass (not used in our study) mulch and their AWCD value under the same cover crop were different from us, which were probably because the soil microbial carbon metabolic capacities in apple orchards change as the trees age and climate. On the other hand, mulching also has different long- and short-term effects on soils’ physical properties, which could be the reason to interpret why there were AWCD value differences between two researches under same cover crops: in our experiment, the year using cover crops were more than that in experiment and physical properties could also affect soil microbial metabolic capacity [8,20].

**Figure 4.**
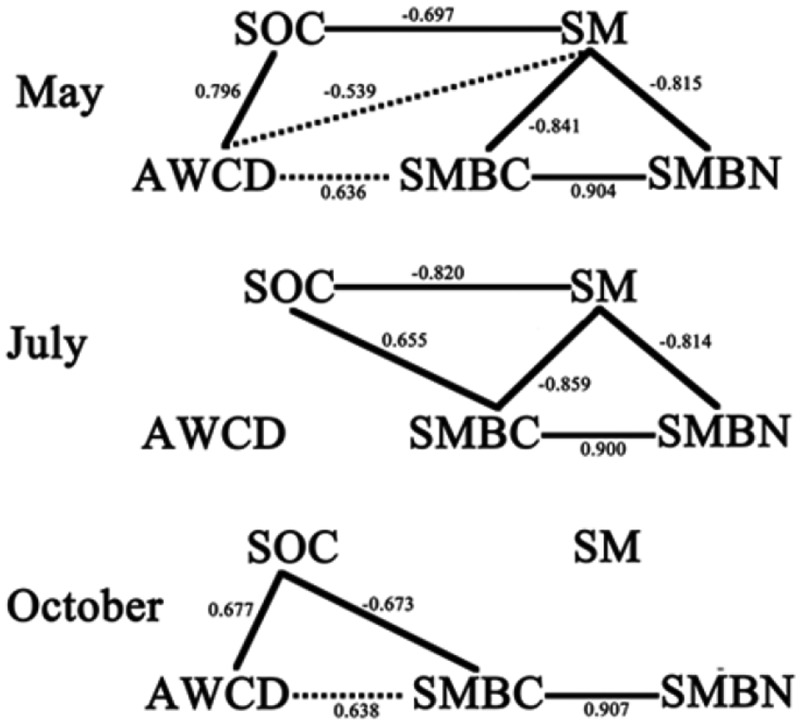
Soil microbial biomass carbon and nitrogen contents (mean ± standard deviation) under indicated cover crops. Note: (1) AW – intercropping of apple and white clover (*Trifolium repens*); (2) AC – intercropping of apple and crown vetch (*Coronilla varia L*.); (3) AS – intercropping of apple and the grass (*Dactylis glomerata L*.); and (4) AH – intercropping of apple and bird’s foot trefoil (*Lotus corniculatus L*.).

The promotion of soil microbial metabolic ability was pronounced in spring and October, and such metabolic capacity changes will have direct net effects on orchard soil nutrient release [23], especially SOC contents. Accordingly, the changes in soil microbial carbon metabolic ability resulted in significant increases in SOC contents (Figure 4), as noted by other scholars [8]. And correspondingly, there were no significant effects on orchard SOC contents under CT treatment as metabolic capacity changes. These outcomes, together with the correlation between AWCD and SOC in Figure 3, show that the promotion of soil microbial metabolism is probably an important mechanism for cover crops to improve soil nutrient accumulation, especially carbon accumulation. However, unlike microbial metabolic capacity, AC rather than AW had the strongest SOC-enhancing effect, which probably illustrated that soil microbial Carbohydrates metabolism was very important in the accumulation of SOC.

There were also significant between-season differences of iCLPPs results in our experiments, which have been also reported in the research [9] found seasonal effects through CLPPs data but did not find significant between-treatment differences, which probably because their experiment only established under two land-use types: apple orchard and boundary bush, and we established our experiment under two different soil management measures: clean CT (monoculture) and cover cropping (intercropping). In fact, there were no significant between-season differences under clean CT in apple orchard soil, which illustrated that these between-season differences could be related to using plants or the amount of using plants. So, the interaction caused by intercropping was probably an important reason leading to the differences in soil microbial carbon metabolism. The interaction caused by intercropping has been confirmed to increase land equivalent ratios [24] and promote nitrogen [25] and phosphorus [26] utilization when there were only few studies focus on the carbon change caused by intercropped interaction.

Root exudate was an important mechanism for intercropped interaction and cover crops to increase soil nutrient level [27], which could also explain different SOC content under different cover crops in the present study. Generally, the legumes (AW, AC and AH) promoted soil microbial carbon metabolism more effectively than the grass (AS), in accordance with previous findings. The decomposition of litter and root exudates from planted legumes may provide soil microorganisms with large amounts of Carbohydrates, Amino acids, Carboxylic acids and other carbon substances [28]. In addition, several studies have shown that root exudates also provide carbon and nitrogen substances for microbes, and both their species and quantities influence the growth, reproduction, metabolism and hence abundance (relative and absolute) of various groups of soil microorganisms. So, AS treatment had the lowest SOC-enhancing effect compared to other cover crops and different cover crops had different effects on soil microbial carbon metabolism because there were different root exudates released in different time. And according to Table 3, the increase of Richness index and the decrease of Evenness index showed that covering crops not only affected the number of carbon substances that soil microbes could metabolize, but also affected the types of soil microbial preferential metabolism, which could also be explained by different root exudates.

Besides the effects caused by root exudates, there were also many environmental factors could be affected by cover crops using like soil moisture, physical properties, microbial diversity [2,4,13,29,30] and thus affected soil microbial carbon metabolism. The results also showed that bacterial communities were highly active in summer, which were different from our experiment [9]. In summer, the living mulches had little effect on the soil microbial carbon metabolic ability, and even reduced many of them. This was probably because low soil moisture is often a limiting factor for microbial activity on the Loess Plateau, due to its semi-arid or arid climate, and the water competition between the living mulch and the fruit trees was widely concerned by researches because this competition is one of the important factors that need to be considered for living mulch technology, especially in arid and semi-arid regions [31]. In the present study, cover crops significantly decreased soil moisture in the 0–40 cm layer in July (Table 1). Thus, there are clear risks that cover crops’ competition with the trees for water in apple orchards (or other agricultural systems) will significantly reduce soil moisture contents, which was consistent with findings of other researchers [32], and hence soil microbial carbon metabolism in the summer. The correlation between soil moisture and other factors in Figure 3 also supported this view.

However, there was no correlation between soil moisture and AWCD in July in Figure 5 when the inconsistency also occurs in other microbial indices such as between Biolog and T-RFLP analyses [8]. In the present study, SMBC and SMBN reflecting soil microbial quantity showing in Figure 4 also showed the inconsistency with CLPPs data, which illustrated that in July soil microbes under cover crops had larger amounts and weaker metabolic activities than under clean CT. So, we supposed that soil microbes probably metabolized other carbon substances because of soil water deficiency in July to maintain the large quantity and these carbon substances could not be determined by Eco-Biolog Plates. Thus, CLPPs data showed that cover crops significantly inhibited soil microbial carbon metabolism.

## Conclusion

Soil management using cover crops could significantly improve soil microbial carbon metabolic capacity, but this promotion could be inhibited by other factors. Notably, the vigorous activities and water demand of cover crops could lead to soil moisture shortages, especially in arid or semi-arid regions, resulting in the reduction or the transition of soil microbial metabolic activities. In orchards, soil water reductions may have more serious effects than reductions of soil microbial metabolic activities. Thus, we should fully consider the local climate and physiological characteristics of cover crops when using cover crops in orchards to avoid this disadvantage. In this study, four treatments involving cover crops all had negative impacts on soil moisture in the apple orchard on the Loess Plateau in summer, reducing soil moisture and soil measured microbial metabolic activities. Soil moisture contents also declined in spring, but not sufficiently to inhibit soil microbial metabolism.

Moreover, even in spring and fall, the four cover crops had different effects on soil microbial carbon metabolic capacity, presumably due to differences in root exudates between the cover crops, and strongly differing seasonal variations associated with their physiological activities. Cover crops probably introduce additional carbon sources to the soil, which reflect in that cover crops affected the number of carbon substances that soil microbes could metabolize and the types of soil microbial preferential metabolizing. The promotion of soil microbial carbon metabolic activities caused by cover crops was probably an important mechanism of cover crops to improve soil nutrient, especially soil organic content when cover crops significantly promoted SOC accumulation, and there was a significant positive correlation between SOC and AWCD. Finally, legumes showed more powerful SOC-enhancing ability than grass and were very meaningful for improving soil quality and soil carbon accumulation.

## Highlights

- Living mulch can change soil microbial metabolism mediated through root exudates
- Seasonal root exudates emission led to soil microbial metabolism seasonal change
- The water competition between ground cover and crop should be considered
